# Electronic-Sports Experience Related to Functional Enhancement in Central Executive and Default Mode Areas

**DOI:** 10.1155/2019/1940123

**Published:** 2019-01-22

**Authors:** Diankun Gong, Weiyi Ma, Tiejun Liu, Yuening Yan, Dezhong Yao

**Affiliations:** ^1^The Clinical Hospital of Chengdu Brain Science Institute, MOE Key Lab for Neuroinformation, University of Electronic Science and Technology of China, Chengdu, China; ^2^School of Life Science and Technology, Center for Information in Medicine, University of Electronic Science and Technology of China, Chengdu, China; ^3^School of Human Environmental Sciences, University of Arkansas, Fayetteville AR 72701, USA

## Abstract

Electronic-sports (e-sports) is a form of organized, online, multiplayer video game competition, which requires both action skills and the ability and process of forming and adapting a strategy (referred to as strategization hereafter) to achieve goals. Over the past few decades, research has shown that video gaming experience has an important impact on the plasticity of the sensorimotor, attentional, and executive brain areas. However, little research has examined the relationship between e-sports experience and the plasticity of brain networks that are related to strategization. Using resting-state fMRI data and the local functional connectivity density (lFCD) analysis, this study investigated the relationship between e-sports experience (League of Legends [LOL] in this study) and brain plasticity by comparing between top-ranking LOL players and lower-ranking (yet experienced) LOL players. Results showed that the top-ranking LOL players had superior local functional integration in the executive areas compared to lower-ranking players. Furthermore, the top-ranking players had higher lFCD in the default mode areas, which have been found related to various subprocesses (e.g., memory and planning) essential for strategization. Finally, the top-ranking players' lFCD was related to their LOL expertise rank level, as indicated by a comprehensive score assigned by the gaming software based on players' gaming experience and expertise. Thus, the result showed that the local functional connectivity in central executive and default mode brain areas was enhanced in the top-ranking e-sports players, suggesting that e-sports experience is related to the plasticity of the central executive and default mode areas.

## 1. Introduction

One of the most prominent changes to our modern lives is the use of computers and Internet, which has changed our entertainment experience with the introduction of electronic-sports (e-sports). E-sports can take the form of organized, multiplayer video game competition, which may require less infrastructure preparation and team development than traditional team sports (e.g., basketball and football). Thus, e-sports is becoming increasingly popular worldwide across a wide age range [1]. This is demonstrated by the rapidly growing number of e-sports players in recent years [2]. For example, a survey conducted in 2014 showed that League of Legends (LOL, a typical, popular form of e-sports) was played by over 67 million people per month, 27 million people per day, and over 7.5 million people concurrently during peak hours (http://blogs.wsj.com/digits/2014/01/27/player-tally-for-league-of-legends-surges/). E-sports is also gaining increasing research attention given its adaptive effect on human development, since e-sports can be physically and cognitively demanding just like traditional sports. Thus, e-sports can be an important platform for studying brain plasticity [3] and an effective tool for educational and cognitive intervention [4]. This study examines the influence of e-sports experience on neural plasticity.

E-sports may owe its origin to single-player action video games (e.g., Super Mario Bros, Tetris, and Unreal Tournament 2004 [4, 5]), which are mostly sensorimotor and attentional tasks such as avoiding obstacles by pressing keys or aiming and shooting at targets using a mouse [6]. With the development of electronic and Internet technologies, e-sports is now a form of online, organized, multiplayer video game competition, which requires both sensorimotor and strategization (e.g., tactics, logistics, and cooperation with teammates) skills just like traditional team sports. Furthermore, the strategization component may be the core, defining feature of both e-sports and traditional team sports, as strategization is essential for team sports.

A significant and growing body of research has examined the influence of e-sports experience on human development, most of which focuses on the effect of action video gaming (AVG) experience on human sensorimotor and attentional development. For example, behavioral research showed that AVG experience promoted primary cognitive processes (e.g., visual processing [7–9], hand-eye coordination [10], contrast sensitivity [8], oculomotor performance [11], and body movement [12]) and certain higher-level cognitive functions (e.g., selective attention [13], spatial distribution of visuospatial attention [14], attentional capture [15], attention shifting [16], and visual short-term and working memory [17–19]). In addition, neuroscience research found that AVG experience modulated event-related potentials and electroencephalography power, which may be related to AVG experts' superior performance in inhibition responses and cognitive control [20, 21].

The present study examined the relationship between e-sports experience and neural plasticity. LOL—a popular action real-time strategy game—was used in this study [22–24]. A typical LOL gaming session has two competing teams each consisting of five players. Each team controls one virtual champion (a virtual character), who has a set of skills to support his/her team in occupying and protecting battlefields. Players use mice and keystrokes to deploy the champion in order to acquire and defend battlefields and resources. More importantly, players also constantly make and adjust their strategy and tactics according to the status of the battlefield. Thus, LOL requires both action and strategization skills. Using resting-state fMRI data and the diffusion tensor imaging analysis, research found that compared with LOL amateurs, LOL experts had enhanced functional and structural connectivity in the attentional and sensorimotor networks [25–27], thus demonstrating the influence of e-sports on sensorimotor and attentional development. However, little research has examined the relationship between e-sports experience and the plasticity of brain networks that are related to planning and strategization.

This study examined this issue by comparing between top- and lower-ranking LOL players. Based on one's skill level, an LOL player can be either (i) a lowest-ranking player (below the 30^th^ percentile) who is still learning the basic rules of the game, (ii) a lower-ranking but experienced player (between the 51 and 82.1 percentiles), or (iii) a top-ranking, expert player (above the 98.33 percentile) who often focuses on planning and implementing the strategy and tactics in LOL gaming. A player's ranking data are assigned by the LOL software based on the player's experience and expertise compared against the average level across all players worldwide. The ranking data used in this study are available on http://www.laoyuegou.com. This study focused on the top- and lower-ranking LOL players with the lowest-ranking players excluded, because they are still learning the basic rules of LOL. Therefore, any neural plasticity observed in them may be driven by their learning of the basic rules of LOL rather than the acquisition of strategization skills. We predict that compared with the lower-ranking players, the top-ranking players should have improved development in (i) the brain areas related to sensorimotor and cognitive control, which supports their superior action skills in e-sports, and (ii) the default mode network (DMN), which supports long-term memory and planning and thereby is essential for strategization [28].

This study used a cross-sectional design to compare top- and lower-ranking LOL players by analyzing their local functional connectivity density (lFCD) based on resting-state fMRI data and their four-dimensional consistency of local neural activities (FOCA), as research shows that the resting-state brain function is adaptable according to age [29] and learning experience [30]. In addition, lFCD and FOCA are data-driven measures for local changes of brain functions [28, 29]. For example, research found that lFCD can quantify local degree and the size of the local network cluster functionally connected to a brain network node [31]. In addition, lFCD changes according to local energy utilization [31], brain aging [32], and stimulant drugs [33].

## 2. Materials and Methods

### 2.1. Participants

The participants were 26 top-ranking players (mean age = 25.35 years ± 2.39, all male), who were among the top 1.77% players among all LOL players worldwide according to the aforementioned ranking, and 34 lower-ranking but experienced players (mean age = 24.59 years ± 2.13, all male) who were ranked between the 51 and 82.1 percentiles. The two groups were matched in age, IQ assessed through Ravens Progressive Matrices (mean_top_ = 90.1 ± 10.13, mean_lower_ = 89.85 ± 9.57 (Here, we reported the original Raven Matrices Scores, which were not converted into the standard IQ score. A Raven Matrices Score of 90 means that the participant's score is at the 90 percentile.)), and weekly physical exercise time (mean_top_ = 2.32 hours ± 1.2, mean_lower_ = 2.14 hours ± 0.78). All participants were right-handed according to the Edinburgh Inventory [34], had normal or corrected-to-normal vision and normal hearing, and did not have a history of neurological illnesses. All participants accepted the protocol that was approved by the research ethics committee of the University of Electronic Science and Technology of China (UESTC). The study complied with the ethical standards outlined by the Declaration of Helsinki.

### 2.2. Data Acquisition

Images were acquired on a 3T MRI scanner (GE Discovery MR750). Resting-state functional MRI data were acquired using gradient-echo EPI sequences (repetition time [TR] = 2000 msec, echo time [TE] = 30 msec, flap angle [FA] = 90°, matrix = 64 × 64, 3 × 3 × 3 mm voxels, field of view [FOV] = 24 × 24 cm^2^, and slice thickness/gap = 4 mm/0.4 mm), with an eight channel-phased array head coil. All participants underwent a 510 sec resting-state scan that yielded 255 volumes (32 slices per volume).

### 2.3. Functional MRI Data Preprocessing

Functional MRI data preprocessing followed typical preprocessing procedures using SPM8 (Welcome Department of Cognitive Neurology, London, UK) and NIT1.2 (Neuroscience Information Toolbox, http://www.neuro.uestc.edu.cn/NIT.html). These procedures included discarding the first five volumes of each run, slice scan time correction, head motion correction, and image normalization using an EPI template from the Montreal Neurological Institute (MNI) atlas space. Temporal filtering (band-pass) was between 0.01 and 0.08 Hz, and the mean signal was removed.

### 2.4. Local Functional Connectivity Density (lFCD)

The calculation of lFCD followed the standard data analysis procedure used in previous research [31, 35]. For a given voxel *x*_0_, its lFCD was calculated using a searching algorithm which computed the Pearson correlation coefficient (*r*) between *x*_0_ and the closest neighboring voxels. The correlation threshold of *R* = 0.6 was used following the standard data processing procedure [31]. If *r*_0j_ was greater than *R*, *x*_*j*_ was then added to the list of functionally connected voxels. This procedure of data calculation was repeated for the next neighbors in the list. When no new neighbors were available, the number of elements in the list of neighbors (*k*) was defined as the lFCD of *x*_0_. Then, the calculation was initiated for another *x*_0_. This procedure of data calculation was performed for all *x*_0_ voxels. Finally, the individual lFCD maps were normalized to the mean value of each individual map and then spatially smoothed using an FWHM of 8 mm.

### 2.5. Correlation Analyses and Between-Group Comparisons

To investigate whether the brain enhancement observed in the top-ranking players was correlated with their e-sports experience, we examined Pearson correlations between the lFCD and the rank level—a comprehensive score assigned by the gaming software based on players' gaming experience. Multiple comparisons were corrected according to the FDR with *p* < 0.05 and a cluster threshold *k* > 20.

## 3. Results

### 3.1. lFCD Results

**Figure 1:**
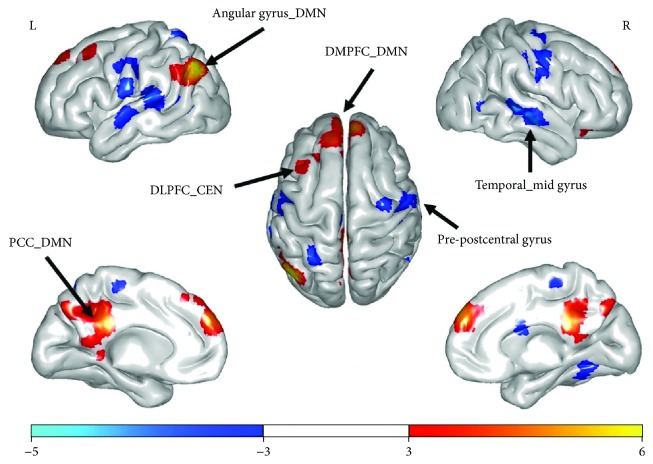
Results of lFCD analyses (*p* < 0.05, FDR-corrected, cluster threshold *k* > 20). Colors ranging from red to yellow (from soft to dark blue) indicate significantly increased (decreased) lFCD in the top-ranking players compared with the lower-ranking players. DMPFC = dorsomedial prefrontal cortex, PCC = posterior cingulate cortex, DLPFC = dorsolateral prefrontal cortex, CEN = central executive network, and DMN = default mode network.

Comparative analyses showed that the top-ranking players had a significantly higher level of lFCD in the integrative brain regions (i.e., DLPFC and PCC) than the lower-ranking players. However, an opposite pattern of results emerged in the primary input-output regions (i.e., precentral gyrus and postcentral gyrus). See Figure 1 and Table 1 for the results.

### 3.2. Correlational Analyses

**Figure 2:**
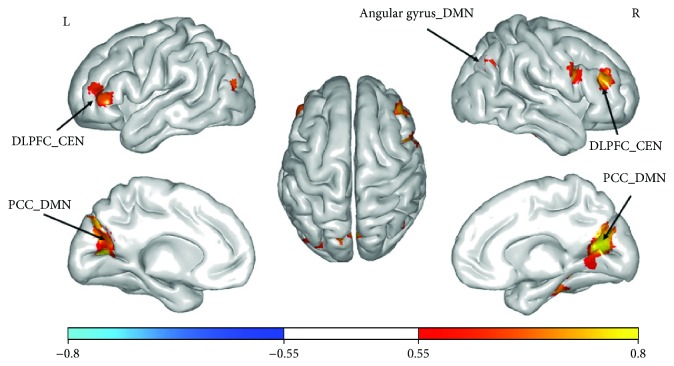
Correlational analyses (*p* < 0.05, FDR-corrected, cluster threshold *k* > 20). Colors ranging from red to yellow indicate *r* values from 0.55 to 0.8 between the rank level and lFCD in the top players. DLPFC = dorsolateral prefrontal cortex, PCC = posterior cingulate cortex, CEN = central executive network, and DMN = default mode network.

Among the top-ranking players, there was a significant, positive correlation between their rank level and lFCD in the brain regions (e.g., DLPFC and PCC) that are responsible for higher-level cognitive functions such as analyzing and integrating information from different sources. However, there were no significant associations in the primary brain regions. See Figure 2 and Table 2 for the results. The results of FOCA and correlation analyses are similar to the lFCD results. See Supplementary Figures 1 and 2.

## 4. Discussions

### 4.1. Enhanced Local Functional Integration in Executive Areas

This study found that the top-ranking players had increased lFCD in bilateral DLPFC compared to the lower-ranking players. Furthermore, among the top-ranking players, their degree of increase in lFCD was associated with their LOL ranking data assigned by the gaming software. This finding suggested that the top-ranking players' LOL experience was related to the plasticity of lFCD. The increase in lFCD may indicate an enhanced local functional integration, supporting the top-ranking players' advanced skills in LOL gaming. Furthermore, the current findings suggest that bilateral DLPFC may be important for the top-ranking players' acquisition of e-sports expertise. Since bilateral DLPFC is essential for central executive network (CEN), the enhanced lFCD observed in bilateral DLPFC may support the top-ranking players' superior integration of CEN, which can improve various subprocesses including updating information, shifting attention, and inhibiting responses, facilitating cognitive control, and executive function [36, 37]. These cognitive processes are essential for LOL gaming during which players need to (1) constantly update information from multiple sources including their champions, teammates, and opponents; (2) frequently shift their attention between their champions and other types of information which are of interest and importance (e.g., location of opponents and teammates and alarm signaled by teammates); and (3) ignore information that is irrelevant to the goal of LOL gaming. Thus, the top-ranking players may have superior CEN integration, cognitive control, and executive function. This is consistent with previous MRI research findings that expertise in AVG and e-sports was associated with increased gray matter volume and enhanced functional integration in DLPFC [5, 25]. Furthermore, research suggested that AVG training could enhance midline frontal theta power in older adults and that the degree of enhancement could predict the older adults' executive function [38].

### 4.2. Enhanced Local Functional Integration in Default Mode Areas

This study also found that the top-ranking LOL players had enhanced local functional integration in *default mode areas*, including the bilateral PCC, parahippocampal gyrus, and right angular gyrus. Furthermore, the enhancements observed in the top-ranking players' DMN areas were also related to their rank level assigned by the LOL gaming software—an objective reflection of the players' gaming experience and expertise (also see supplementary results for details of the results of DMN using FOCA analysis). Thus, the findings revealed a relationship between e-sports experience and the plasticity of default mode areas. Nevertheless, research has demonstrated that DMN is an intrinsic network that tends to be activated in the resting-state but deactivated during the cognitive task [39]. Unlike previous research that showed that DMN often competes against CEN for cognitive resources [37, 39, 40], the current study revealed a positive relationship between the development of DMN and CEN. However, it should be noted that there is also evidence suggesting that DMN can be activated in tasks that require thinking about oneself and others, memory consolidation, and planning [28]. For example, research found that the activation of DMN facilitated the performance of CEN in tasks where the function of CEN needed information preprocessed by DMN (e.g., the face information stored in the long-term memory [41]). Thus, DMN may assist CEN in retrieving information from the long-term memory. In addition, based on the interview of the participants conducted prior to the present study, the top-ranking players tended to prioritize the strategization skills while the lower-ranking players tended to prioritize the action skills. Thus, the top-ranking players believed that the most important skills for LOL gaming were assessing the opponents' psychological status, predicting their actions, and making the team strategy and tactics. These cognitive processes are highly related to DMN [42]. However, the lower-ranking players often believed that the most important skills for LOL gaming were action and sensorimotor skills, which are highly related to CEN [43–45].

Nevertheless, this study focuses on the relationship between LOL experience and the plasticity of *local* connectivity. Our analyses of the global connectivity measures (e.g., global FCD) did not reveal significant results depending on the participants' LOL experience, which may suggest that LOL experience facilitates the specific brain networks, which are closely related to LOL gaming, more than the global brain connectivity.

## 5. Conclusions

This study investigated the relationship between e-sports experience and neural plasticity. Results showed that compared with the lower-ranking players, the top-ranking players had a higher level of lFCD in the executive areas, which may be related to their superior action skills in e-sports. More importantly, the top-ranking players also had a higher level of lFCD in the DMN compared with the lower-ranking players. The enhancement of DMN may be related to their advanced strategization skills in e-sports. To our knowledge, the present study is the first to show that e-sports experience is related to enhanced local functional integration in default mode areas.

## Figures and Tables

**Table 1:** The results of lFCD analyses. The positive (negative) *t* values indicate significantly increased (decreased) lFCD in the top-ranking players compared with the lower-ranking players.

Clusters	Brain regions (AAL template)	Voxels	Peak *t* value	Peak MNI coordinate [*x* *y* *z*]
1	Left superior frontal gyrus	70	3.93	-12 48 48
	Left middle frontal gyrus (DLPFC)	61		
	Left superior frontal gyrus, medial	41		

2	Left superior frontal gyrus (DMPFC)	118	4.65	9 60 39
	Right superior frontal gyrus (DMPFC)	70		

3	Left angular gyrus	142	5.85	-48 -75 39

4	Left precuneus (PCC)	129		0 -39 30
	Right precuneus (PCC)	69	4.2	
	Posterior cingulate gyrus (PCC)	54		

5	Right postcentral gyrus	171	-3.99	27 -21 42
	Right precentral gyrus	128		

6	Left middle temporal gyrus	175	-4.72	-39 -60 6
	Left superior temporal gyrus	162		
	Left postcentral gyrus	148		

7	Right superior temporal gyrus	125	-4.1	57 -15 -9
	Right middle temporal gyrus	39		

**Table 2:** Correlational analyses between the lFCD and the rank level among the top-ranking players.

Clusters	Brain regions (AAL template)	The number of voxels	Peak *r* value	Peak MNI coordinate [*x* *y* *z*]
1	Parahippocampal gyrus	23	0.7	36 -27 -24
2	Left middle frontal gyrus (DLPFC)	27	0.66	-51 36 3
3	Left precuneus (PCC)	38	0.88	-15 -72 15
4	Right precuneus (PCC)	87	0.82	15 -66 15
5	Right middle frontal gyrus (DLPFC)	44	0.72	48 39 24
6	Right angular gyrus	49	0.83	36 -57 30

## Data Availability

Data are available upon request to DKG.

## Supplementary Materials

Supplementary Materials In the computation of the FOCA section, the calculation method and formula of FOCA are detailedly introduced. Supplementary Figure 1 showed the results of FOCA analysis. Supplementary Figure 2 showed the correlation analysis between the game levels and FOCA in the top players. Supplementary Figure 3 showed the distribution of the top-ranking players' rank scores.
