# Supplementary material for: Electronic-Sports Experience Related to Functional Enhancement in Central Executive and Default Mode Areas
Source: Neural Plast. 2019 Jan 22;2019:1940123. doi: 10.1155/2019/1940123 (PMC6362490; doi:10.1155/2019/1940123)
Supplement: Supplementary Materials — In the computation of the FOCA section, the calculation method and formula of FOCA are detailedly introduced. Supplementary Figure 1 showed the results of FOCA analysis. Supplementary Figure 2 showed the correlation analysis between the game levels and FOCA in the top players. Supplementary Figure 3 showed the distribution of the top-ranking players' rank scores. [file 1940123.f1.pdf]

Supplementary materials

The computation of FOCA

For image data, a time series of a given voxel with those of its nearest neighbors (26 voxels) can be obtained, and a *FOCA* value assigned to the given voxel can be calculated by the following procedure. In the temporal correlation aspect, C_t is defined as the mean of the cross-correlation coefficients of the local voxels and is given in the equation:

$$C_t = \left| \frac{\sum_{i<j}^{N} r_{ij}^t}{N} \right| ; N = \frac{k(k-1)}{2}$$

where r_{ij}^t is Pearson's correlation coefficient between voxel i and voxel j, and k is the number of voxels in the local region (27 voxels). On the other hand, for the local spatial distribution in the *m*-th timepoint, a spatial correlation between distributions in the neighboring time point is defined as following:

$$\bar{r}^5{}_m = \frac{r^5{}_{m,m-1} + r^5{}_{m,m+1}}{2}$$

where *r* is Pearson's correlation coefficient and m is the *m*-th timepoint. The mean spatial correlation (C_s) across all timepoints is given in the equation:

$$C_s = \left| \frac{\sum_{m=1}^{N_t} \bar{r}^5{}_m}{N_t} \right|$$

where N_t is the number of time points. Then (Fig. 1c), the *FOCA* value is defined as:

$$FOCA = C_t * C_s$$

Finally, the *FOCA* value for every voxel can be calculated by Eq. (4) to form *FOCA* maps. In order to reduce the effect of individual variability, we also normalized the *FOCA* value of each voxel by dividing it by the mean *FOCA* of the whole brain for each subject, that is:

$$FOCA_{norm} = \frac{FOCA}{mean(FOCA)}$$

In addition, based on the aforementioned definition of *FOCA*, a simulation was designed to explain the hypothesis of spatiotemporal consistency in a local region[47].

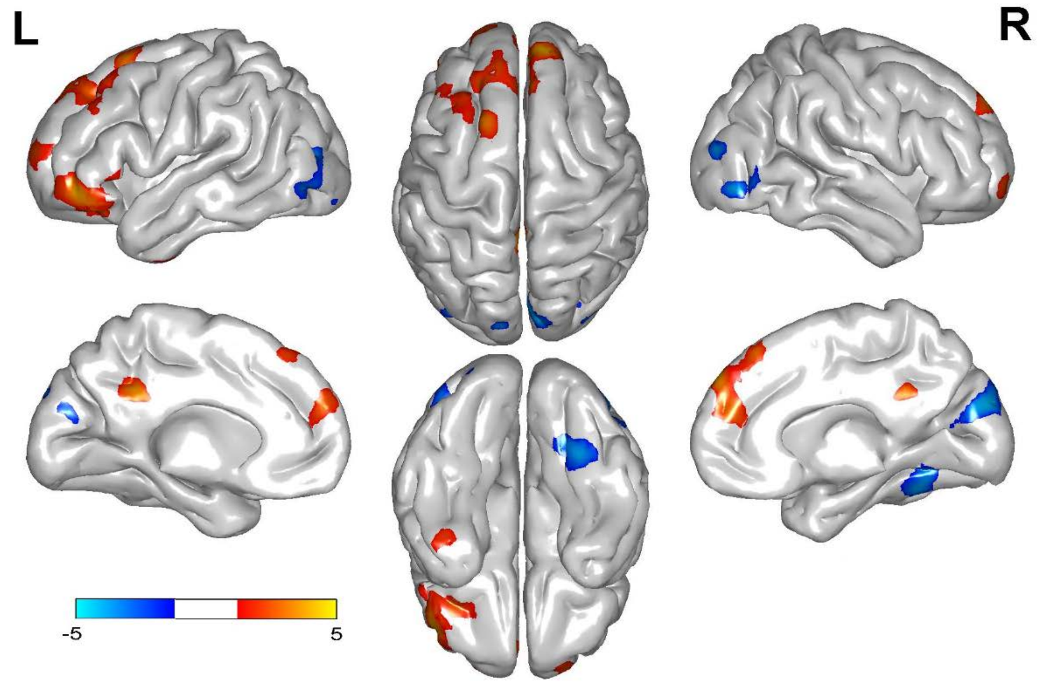

Supplementary Figure 1: FOCA analysis ($p < 0.05$, FDR-corrected, cluster threshold k>20). Colors ranging from red to yellow (from soft to dark blue) indicate significantly increased (decreased) FOCA in the top players than the controls.

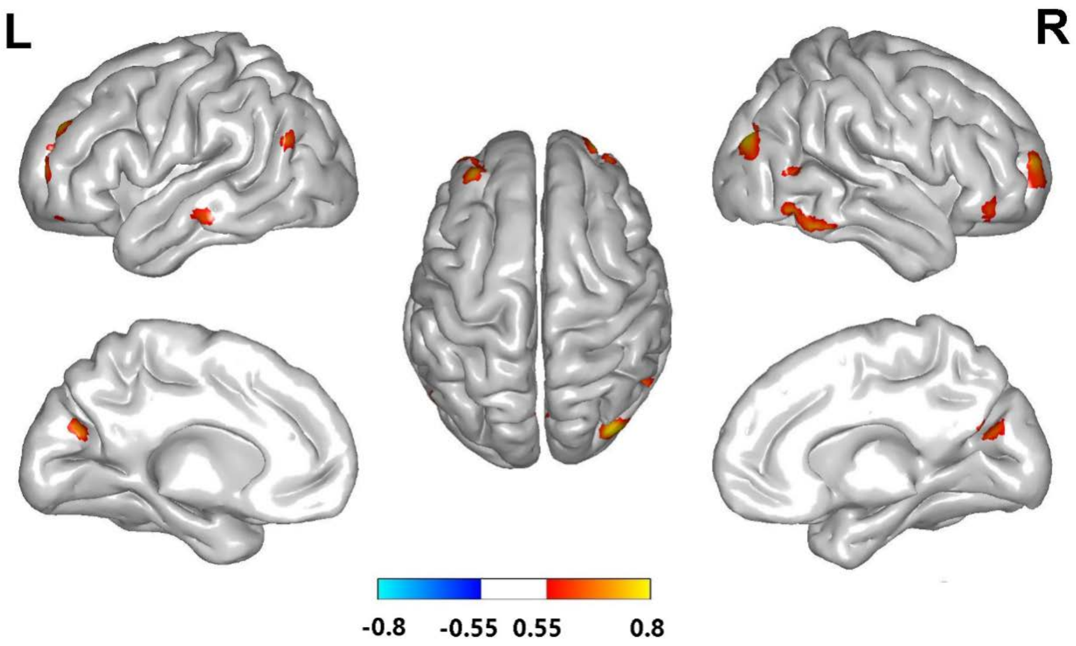

Supplementary Figure 2: Correlation analysis ($p < 0.05$, FDR-corrected, cluster threshold $k > 20$). Colors ranging from red to yellow indicate *r*-value from 0.55 to 0.8 between the game levels and FOCA in the top players.

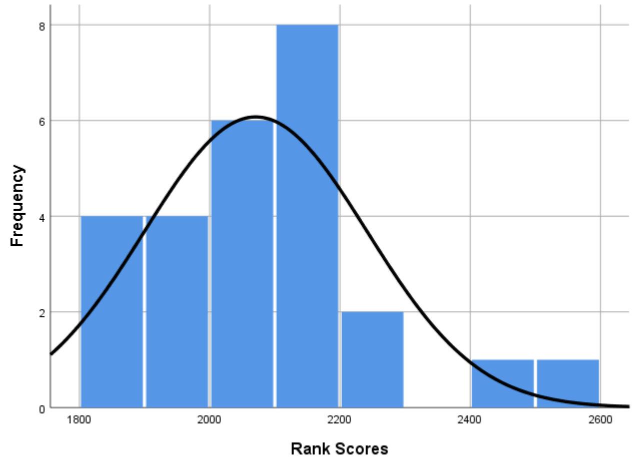

Supplementary Figure 3: The distribution of the top-ranking players’ rank scores.
